# Design of a Deep Learning-Based Metalens Color Router for RGB-NIR Sensing

**DOI:** 10.3390/nano14231973

**Published:** 2024-12-08

**Authors:** Hua Mu, Yu Zhang, Zhenyu Liang, Haoqi Gao, Haoli Xu, Bingwen Wang, Yangyang Wang, Xing Yang

**Affiliations:** State Key Laboratory of Pulsed Power Laser Technology, College of Electronic Engineering, National University of Defense Technology, Hefei 230037, China; muhua05@nudt.edu.cn (H.M.); liangzy21@nudt.edu.cn (Z.L.); gaohaoqi77@nudt.edu.cn (H.G.); xuhaoli@nudt.edu.cn (H.X.); wbw@nudt.edu.cn (B.W.); wangyy@nudt.edu.cn (Y.W.)

**Keywords:** deep learning, metalens, color filter, CMOS image sensor

## Abstract

Metalens can achieve arbitrary light modulation by controlling the amplitude, phase, and polarization of the incident waves and have been applied across various fields. This paper presents a color router designed based on metalens, capable of effectively separating spectra from visible light to near-infrared light. Traditional design methods for meta-lenses require extensive simulations, making them time-consuming. In this study, we propose a deep learning network capable of forward prediction across a broad wavelength range, combined with a particle swarm optimization algorithm to design metalens efficiently. The simulation results align closely with theoretical predictions. The designed color router can simultaneously meet the theoretical transmission phase of the target spectra, specifically for red, green, blue, and near-infrared light, and focus them into designated areas. Notably, the optical efficiency of this design reaches 40%, significantly surpassing the efficiency of traditional color filters.

## 1. Introduction

The development of CMOS Image Sensors (CIS) [1,2] based on silicon photodetectors has garnered significant attention across various optical fields due to their effective capability to capture visible and near-infrared light images. To achieve higher image quality, specifically enhanced image resolution, the most intuitive approach is to increase the number of pixels in the sensor area. However, as the pixel count gradually increases, the pixel size inevitably decreases, leading to a significant reduction in the light-receiving area of each pixel. This reduction in pixel size results in a considerable decrease in the sensitivity and signal-to-noise ratio of the sensor. Traditionally, there are two approaches to address this issue: using back-illuminated (BSI) sensors [3,4] and employing dye-based color filters [5,6]. The former suffers from the problem of larger pixel sizes, while the latter, although capable of providing unparalleled high transmission efficiency (around 90%) and color purity, faces the challenge of lower optical efficiency. In practical applications of this configuration, most of the light is filtered out by the color filters, resulting in a waste of light, with actual optical efficiency being less than 25% for quad-wavelength (Red(R), Green(G), Blue(B), and Near-Infrared(NIR)) color filters or around 33% for tri-wavelength (R, G, B) color filters [7,8]. To address these challenges, researchers have proposed various methods, including color routers [9,10], plasmonic filters [5], and emerging image sensors [11]. Among these, the color router is a technology that can directly separate colors based on the wavelength of the incident light, and it has been validated through approaches such as metalens [8,12,13], metamaterials [7], and photonic crystals [14].

Metalens [15,16,17,18,19], with their potential for free control of light beams, are expected to achieve promising and significant results. A metalens is a two-dimensional array of subwavelength nanostructures that can flexibly manipulate the amplitude [20,21], phase [22], and polarization [23] of incident light. To tackle the aforementioned issues, existing research has demonstrated that dielectric metalens constructed from subwavelength arrays can be designed as nanophotonic color routers, effectively separating the spectrum from visible light to near-infrared and focusing it onto target pixel surfaces at different locations. Chen B H et al. [24] reported a full-color router featuring GaN metalens, which achieves multi-wavelength routing by integrating 4 × 4 spatial multiplexing nanocolumns into a complex unit cell. This design enables the routing of individual primary colors to different spatial positions. Hong Y J et al. [25] present a single-layered metasurface capable of sorting a broad range of light to four spectral channels that the silicon photodetector can respond to. The proposed metasurface-based color router shows 32% averaged optical efficiency over operating RGB and NIR wavelengths. This nanoscale photonic color router based on metalens can provide unique light path trajectories depending on the spectral range of the target. Moreover, since its fundamental principle involves altering the wavefronts of different wavelengths rather than absorption, its optical efficiency remains relatively high. However, during the design process of these metalens, the standard approach [26] typically involves researchers using their experience to design one or several initial basic structures, such as cylinders or rectangular prisms. They then scan the characteristic parameters (e.g., radius, length/width) to obtain the phase and transmittance results corresponding to different structures. Finally, suitable meta-units are selected based on the desired phase distribution and placed in predetermined locations. While this method is relatively straightforward, it involves a repetitive experimental process that requires numerous complex numerical simulations, which can be time-consuming and labor-intensive. Although some studies have integrated genetic algorithms [27,28], particle swarm algorithms [29], and gradient descent algorithms [30] for the design of metalenses, each optimization still necessitates the re-simulation of the properties of the meta-units, further increasing the complexity and duration of the design process.

Deep learning [31,32,33] is a data-driven approach based on deep neural networks (DNNs) that have achieved significant success in various fields, including image recognition, natural language processing, and speech recognition. It can learn complex mapping relationships between inputs and outputs without requiring consideration of intricate physical principles, and it has found critical applications in several areas of nanophotonics. In this study, to efficiently control the directed transmission of red, green, blue, and near-infrared light to the target pixel surfaces of the CIS, we designed a discrete distribution metalens based on a deep learning network, successfully applying it to an RGB-NIR color router. Specifically, we first designed a discrete distribution meta-atom structure to effectively meet the phase combination requirements for different wavelengths. The designed discrete meta-atom structure consists of a 20 × 20 pixel array, where each pixel can be represented by either 0 or 1, with 0 indicating the absence of a pixel structure at that position and 1 indicating its presence. Next, we simulated various discrete meta-atom structures and outputted their transmittance and phase curves to construct a database for deep learning. We then developed a deep learning algorithm based on the transformer structure by incorporating a wavelength embedding method. This deep learning network was trained on the previously obtained database to enhance its prediction accuracy. Finally, we utilized the high-precision forward prediction capability of the deep learning algorithm in conjunction with a particle swarm optimization algorithm to design both the single-wavelength focusing metalens and the color router metalens. The model predicts a wavelength range of 400–1100 nm, encompassing the entire visible light and near-infrared spectrum, which exceeds the prediction range of most existing deep learning approaches. The transmittance prediction error of the model is mainly within the range of 0 to 1%, with an overall average prediction error of 0.5%. And the speed of predicting a single meta-atom is only 1 ms (The network can process 1000 inputs in parallel within 1 s). Building on this, the single-wavelength metalens designed in conjunction with the particle swarm optimization algorithm can achieve near-perfect alignment with the predetermined focal length, with a focusing efficiency of up to 87.2%. The designed color router metalens is capable of routing the corresponding paths of red, green, blue, and near-infrared light to four distinct positions, achieving an average efficiency of 40%, which surpasses the efficiency of traditional color filters.

## 2. Theory and Method

**Figure 1:**
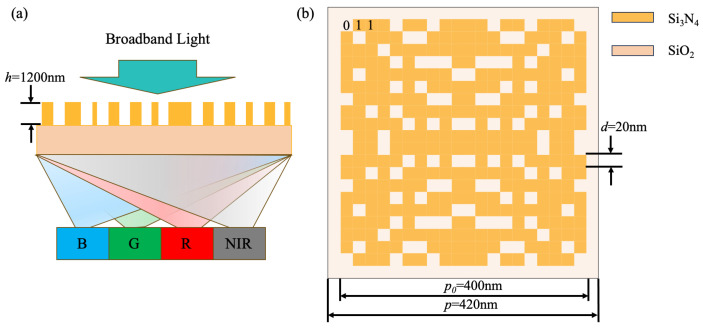
(**a**) The schematic diagram of the RGB-NIR color router. (**b**) The schematic diagram of the meta-atom.

Figure 1 illustrates the schematic diagram of the RGB-NIR color router and the representation of the meta-atoms. As shown in Figure 1a, unlike traditional dye filters that selectively transmit the target spectrum while absorbing the rest, the color router based on the metalens can guide red, green, blue, and near-infrared light into four distinct channels, promising a significant enhancement in optical utilization. In most optical metalens designs, high-refractive-index materials (such as silicon and germanium) are typically chosen to fabricate meta-atoms for easier attainment of the desired phase distribution. However, in this design, due to the target wavelength range of 400–1100 nm, these materials exhibit considerable absorption. Therefore, this study selects silicon nitride (Si_3_N_4_, refractive index obtained from the data measured by Phillip in 1973) as the structural layer for the meta-atoms and silicon dioxide (SiO_2_ refractive index from the Handbook of Optical Constants of Solids by Edward D. Palik) as the substrate layer for the meta-atoms. As shown in Figure 1b, the schematic diagram of the designed discrete meta-atom structure features a period of 420 × 420 nm, with the discrete structure area measuring 400 × 400 nm. The spacing is included to prevent crosstalk between adjacent meta-atoms. The discrete structure consists of a 20 × 20 pixel array, where each pixel can be represented by either 0 or 1, with 0 indicating the absence of a pixel structure at that position and 1 indicating its presence. All pixels have a height of 1200 nm and a side length of 20 nm.

### 2.1. Building Dataset

In this study, we utilized the commercial software Lumerical FDTD 2022 to simulate the meta-atoms and used the results as our dataset. During the simulation process, the simulation region in the *x* and *y* directions was set to periodic boundary conditions, while the *z* direction employed Perfectly Matched Layer (PML) boundaries. The incident light was configured as a plane wave, vertically incident on the surface of the meta-atoms from the positive direction of the *z*-axis. The simulation mesh density was set to 0.2 nm to ensure the accuracy of the results. Subsequently, several random 20 × 20 matrices composed of 0 and 1 were generated. Based on these matrices, the corresponding meta-atom structures were established according to the previously mentioned rules. After the simulations were completed, the matrices, transmittance curves, and phase curves were packaged and input into the deep learning network as a dataset for subsequent training processes. The simulation time for each meta-atom varied, ranging from several seconds to a few minutes. To enrich the dataset as much as possible, we simulated 20,000 different meta-atom combinations, with the entire simulation process requiring approximately 100 h. However, our simulations were conducted on a single computer using multitasking. Distributing the simulation tasks across multiple computers or utilizing a server could significantly reduce the time required for dataset construction.

### 2.2. Establishing a Deep Learning Network Structure

**Figure 2:**
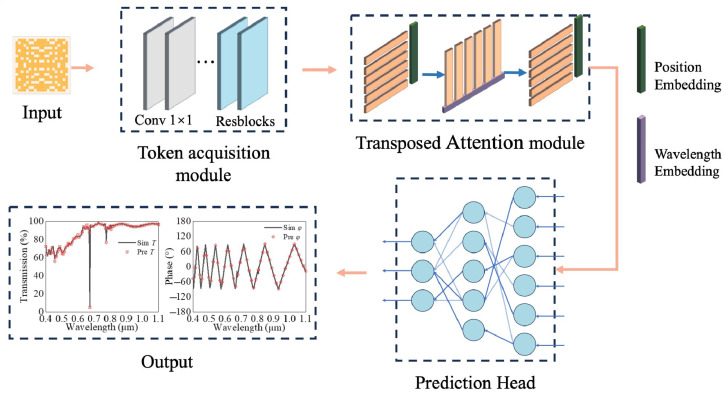
Flowchart of the prediction process for neural network architecture.

Deep learning algorithms typically consist of multiple neural network layers, where each layer transforms the input data into higher-level feature representations through nonlinear activation functions. The basic layer usually includes Multi-Layer Perceptron (MLP), Convolutional Neural Networks (CNNs) [34], and transformer [35], etc. Among these, CNNs are widely applied in the design of various photonic devices and metalenses due to their advantages in automated feature extraction, high expressive power, and robust scalability. To maximize prediction accuracy, we adopted a CNN-based architecture as the core of our model and developed a deep learning algorithm based on the transformer structure by incorporating a wavelength embedding method. The flowchart of this process is illustrated in Figure 2. The specific steps are as follows: (1) Input module: Input the matrix of the discrete metalens along with the transmittance and phase results into the deep learning algorithm. (2) Token generation module: This module involves a detailed extraction of features from the metalens, which can generate the tokens for the input of the Transformer structure. Unlike the traditional token generation method, which usually directly concatenates images with the position embedding information to form the tokens, our approach incorporates multiple 1 × 1 and 3 × 3 convolutional layers to further extract local information. At this point, each token not only carries information about the current pixel, but also surrounding information. (3) Transpose Attention module: This module utilizes the basic network model of the Transformer architecture to extract global features, where the attention mechanism is the core component of the Transformer. Its formula can be expressed as [36]:

(1) $$\mathrm{Attention}\left(Q,K,V\right)=\mathrm{softmax}\left(\frac{QK^{T}}{\sqrt{d_{k}}}\right)V,$$

where *Q*, *K*, and *V* are the input vectors representing the query, key, and value, respectively, while $d_{k}$ denotes the dimensionality of the vectors. Unlike traditional Transformers that solely use Multilayer Perceptron (MLP) to extract *Q*, *K*, *V* information from tokens, our approach in this paper employs an MLP combined with a CNN using 3 × 3 convolutional kernels to obtain *Q*, *K*, *V* information. This integration enables the original global Transformer to possess local information extraction capabilities, thereby further enhancing its effectiveness. (4) Building the prediction head: Given the wide wavelength range predicted for the meta-atoms, which can reach up to 700 nm, traditional prediction methods are insufficient to meet these requirements. Therefore, we introduce a non-shared parameter prediction head and utilize the Rational activation function to model complex variations across this broad wavelength range. Firstly, the multiple vectors output by the transposed attention structure are reshaped into one vector. Then, we adopt the grouped MLP layers to form the prediction head. The number of groups equals frequency points. Therefore, the prediction results at each frequency point have special parameters, which greatly enhance the nonlinear expression capability. After that, to further enhance the nonlinear expression capability of the prediction head, we introduce the Rational activation function in the grouped MLP layers. The Rational activation function is a type of activation function based on rational functions, typically represented as a fractional polynomial. Its main characteristics include the ability to provide greater flexibility and expressive power by adjusting the polynomials in both the numerator and denominator. This results in superior performance when modeling complex nonlinear relationships. Its functional expression is as follows [37]:

(2) $$\phi \left(x\right)=\frac{P\left(x\right)}{Q\left(x\right)}=\frac{\sum_{i=0}^{r_{P}}a_{i}x^{i}}{\sum_{i=0}^{r_{Q}}b_{j}x^{j}},$$

where $r_{P}$ = deg(*P*(*x*)), $r_{Q}$ = deg(*Q*(*x*)), $a_{i}$ and $b_{j}$ are the trainable parameters within the activation function. Additionally, phase discontinuities can occur at specific wavelengths, leading to phase jumps that may affect prediction accuracy. To address this, we applied sine and cosine operations to the phase values to ensure continuity. Furthermore, we performed logarithmic transformations on the transmittance values, which enhances the ability of the deep learning network to predict subtle changes in the transmittance curves. (5) Output module: This module outputs the predicted transmittance and phase values corresponding to the target meta-atom. The illustration in Figure 2 shows a comparison of the simulated and predicted transmittance and phase curves for a specific meta-atom during the training process.

During the training process, the entire database was randomly divided into three groups: a training set, a validation set, and a test set, with proportions of 70%, 15%, and 15%, respectively. The neural network was trained on a platform consisting of an Intel(R) Core(TM) i7-13700K processor and an RTX 4090 graphics card, with a training duration of approximately 72 h. To ensure that the designed deep learning network could simultaneously predict both phase and transmittance, we selected the Mean Absolute Error (MAE) as the loss function, defined in its basic form as:

(3) $$Loss={\left|\cos\left(\varphi_{pre}\right)-\cos\left(\varphi_{sim}\right)\right|}_{1}+{\left|\sin\left(\varphi_{pre}\right)-\sin\left(\varphi_{sim}\right)\right|}_{1}+\left|\mathrm{lg}T_{pre}-\mathrm{lg}T_{sim}\right|,$$

where $\varphi_{pre}$ and $\varphi_{sim}$ represent the predicted and simulated phase vectors, respectively, and $T_{pre}$ and $T_{sim}$ represent the predicted and simulated transmittance vectors.

### 2.3. Training Results of Deep Learning Models

**Figure 3:**
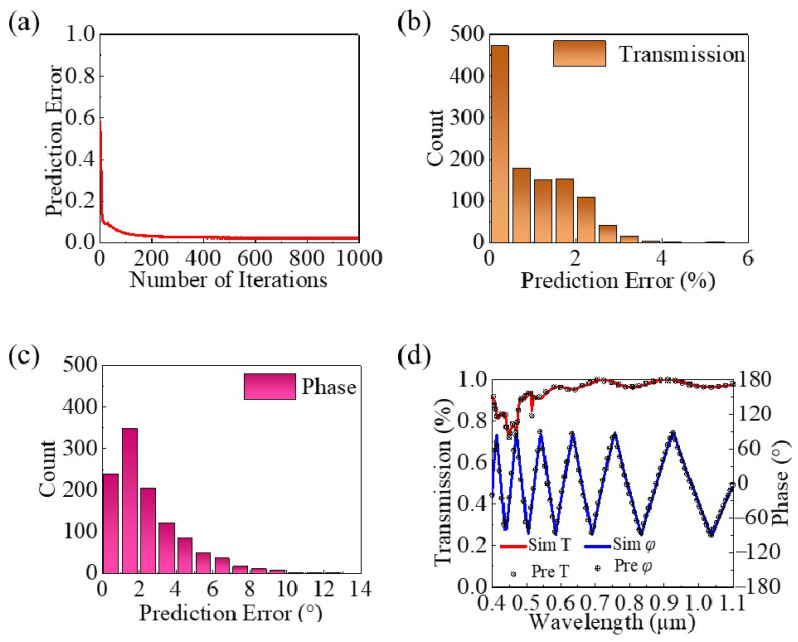
(**a**) Convergence curve of the prediction error for the deep learning network. (**b**) Statistical chart of the transmittance prediction error of the model on the test set. (**c**) Statistical chart of the phase prediction error of the model on the test set. (**d**) Comparison curve of transmittance and phase predictions against simulations for a randomly selected structure in the test set.

As shown in Figure 3, the training results of the deep learning network are presented. Figure 3a indicates that as the number of iterations increases, the error gradually decreases, stabilizing after 300 iterations. This suggests that the prediction accuracy of the deep learning network has reached a stable and extremely high level. Figure 3b,c provide statistical results of the average prediction errors for transmittance and phase on the test set. The transmittance prediction error of the model is mostly within the range of 0 to 1%, with an overall average prediction error of 0.5%. The phase error primarily falls between 0° and 4°, with a total average error of 1.75°. This demonstrates that the predictions made by our model are very close to the actual values.

To more clearly illustrate the prediction performance of the deep learning network on the meta-atoms, Figure 3d presents a comparison between the predicted transmittance/phase curves and the simulated transmittance/phase curves for two randomly structured meta-atoms. As seen in the figures, the predicted data closely aligns with the simulated data, except at a few specific positions, indicating the high accuracy of the predictions made by the designed deep learning network. Moreover, the network can generate nearly 1000 results in 1 s, demonstrating a significant advantage over the time consumed for simulation.

## 3. Design and Analysis of Color Router Metalens

**Figure 4:**
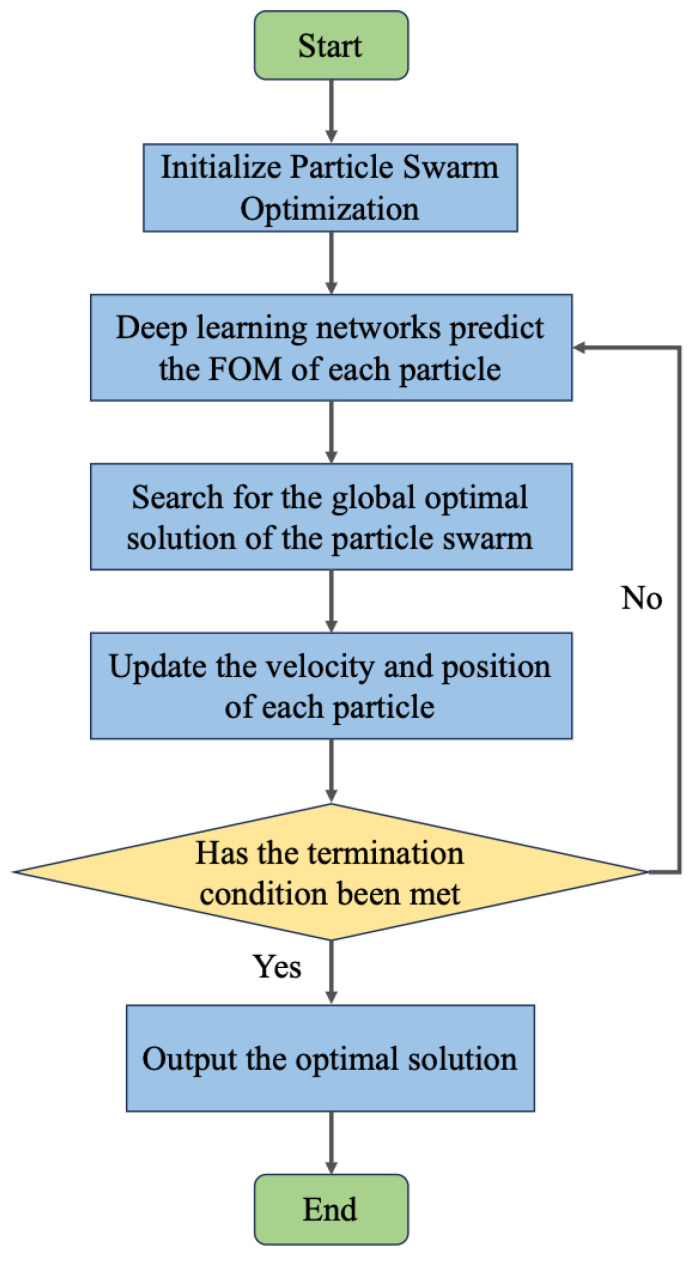
Flowchart of the inverse design process combining deep learning network architecture with particle swarm optimization algorithm.

After completing the steps mentioned above, we proceeded to use the trained deep learning network in conjunction with an inverse design algorithm for the design of the color router. Inverse design is an optimization method aimed at improving existing designs or generating superior new structures to meet specific performance objectives through automated techniques. Standard inverse design algorithms include genetic algorithms (GA) [38], particle swarm optimization algorithms (PSO) [39,40], simulated annealing algorithms (SA) [41], and topology optimization algorithms (TO) [42]. Among these, the particle swarm optimization algorithm is a population-based optimization technique characterized by strong global search capabilities and adaptability. Compared to genetic algorithms and simulated annealing algorithms, it exhibits a faster convergence rate. Therefore, in this study, we chose to combine the particle swarm optimization algorithm with the deep learning network to accomplish the design of the color router. Figure 4 presents the overall workflow for the design process. It is important to note that we utilized the deep learning network to replace the original electromagnetic simulation process. This allowed for a faster computation of the figure of merit (FOM) values, which were then fed back into the PSO algorithm for iterative optimization. Given that the prediction time for a single meta-atom is only 1 ms, the time required for the inverse design method based on the deep learning network is significantly reduced, allowing the majority of the target optimizations to be completed in approximately 5 min. The proposed scheme combining deep learning and particle swarm optimization can design metalenses with arbitrary geometric performance over a wide wavelength range, as long as the phase and transmittance distributions of the target metalens are provided in advance. The operational wavelength range is 400–1100 nm, which exceeds the prediction range of most existing deep-learning approaches. As shown in Table 1, a performance comparison is made between the proposed design scheme and other similar design approaches. Furthermore, this method can predict the results of metalens units with different feature sizes (feature size $d_{0}=N*d$, where *N* is a positive integer) by combining adjacent discrete pixels to accommodate various manufacturing constraints.

### 3.1. Design of Single-Wavelength Focusing Metalens

**Figure 5:**
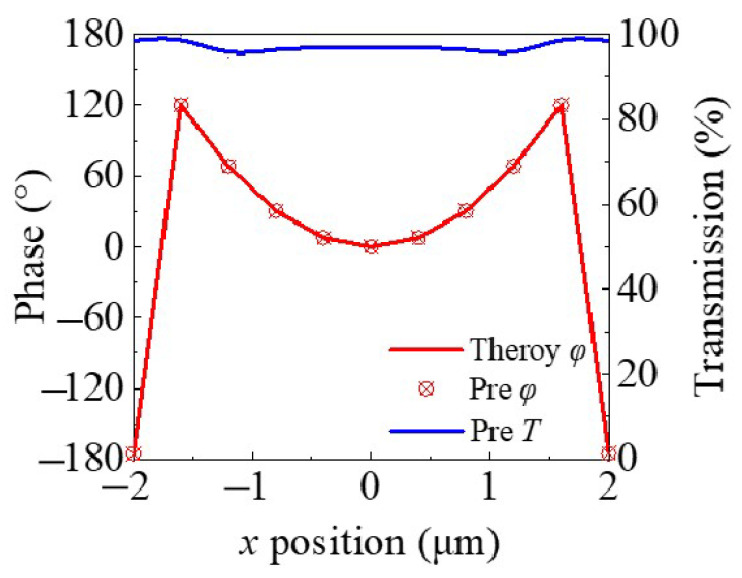
Comparison of the theoretical phase distribution and the phase distribution of the meta-atoms obtained through inverse design, along with the corresponding transmittance curves.

The single-wavelength focusing metalens is an optical element designed based on metasurface principles, tailored explicitly for efficient focusing and imaging at a designated wavelength. Compared to traditional lenses, the single-wavelength focusing metalens offer higher resolution and a more compact form factor, presenting a wide range of potential applications. To preliminarily validate the advantages of combining deep learning networks with inverse design algorithms, we designed a single-wavelength focusing metalens for a wavelength of 750 nm. Initially, we calculated its phase distribution, which can be expressed as:

(4) $$\varphi_{0}\left(x,y\right)=-\frac{2\pi }{\lambda_{0}}\left(\sqrt{x^{2}+y^{2}+f^{2}}-f\right),$$

where (*x*, *y*) represents the center positions of the meta-atoms within the metalens, $\lambda_{0}$ denotes the target wavelength, and *f* indicates the focal length, set at 2 μm. We have established the unit size of the metalens to be 4 × 4 μm. Subsequently, we optimized the meta-atoms using an inverse design approach based on different phase distributions to ensure that the highest transmittance is achieved while meeting the phase distribution requirements. The objective function can be defined as:

(5) $$FOM=\left|\varphi_{n}-\varphi_{0}\right|+\eta \left(1-T_{n}\right),$$

where $\varphi_{n}$ represents the phase of the meta-atoms at the target wavelength during the iterative optimization process, η represents weight, while $T_{n}$ denotes their transmittance. As illustrated in Figure 5, a comparison is made between the theoretical phase distribution and the phase distribution of the meta-atoms obtained through inverse design. For the single-wavelength focusing metalens, it is relatively easy to optimize meta-atoms that are entirely consistent with the theoretical phase. The figure also displays the transmittance corresponding to each phase, with the minimum transmittance reaching 90%. This is highly beneficial for the subsequent focusing efficiency of the metalens.

**Figure 6:**
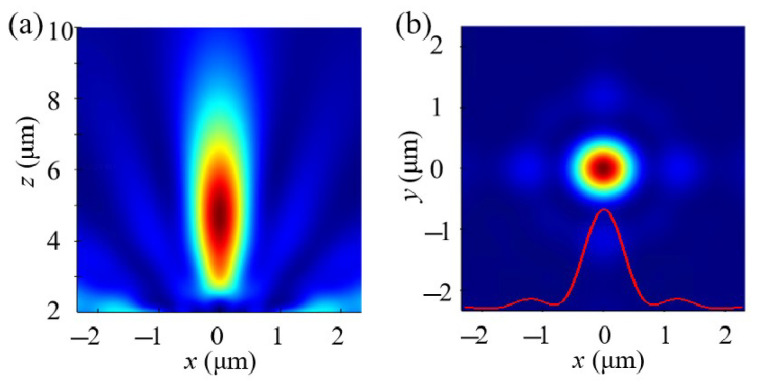
(**a**) The electric field distribution in the focusing cross-section and (**b**) the electric field distribution in the focal plane of the metalens.

Finally, we modeled and simulated the metalens based on the relationship between position and phase. Figure 6 presents the electric field distribution in the focusing cross-section and the electric field distribution in the focal plane of the metalens. From the figures, it can be observed that the designed metalens achieve a focal length of 4.6 μm, with slight deviations from the theoretical value attributed to the diffraction caused by the thickness of the metalens themselves. The full width at half maximum (FWHM) reaches 0.77 μm, fully attaining the diffraction limit level (d=0.5λ/NA). In the focal plane distribution, it is evident that, aside from the vicinity of the focal point, there is almost no interference from stray light at other positions. We calculated the focusing efficiency, which reached 87.2%. Focusing efficiency is defined as the ratio of the power within the focal region (a circle with a radius of three times the FWHM) to the total transmitted power, marking a significant achievement in the performance of the metalens.

### 3.2. Design of Color Router Metalens

The color router needs to focus light at predetermined positions based on different wavelengths, which is equivalent to establishing a metalens that can simultaneously satisfy four distinct phase distributions for the red, green, blue, and near-infrared bands. The corresponding focal positions for the four bands set at (−1 μm, −1 μm), (−1 μm, 1 μm), (1 μm, −1 μm), and (1 μm, 1 μm), respectively. Thus, we first calculated the phase distributions for the four wavelengths, which can be expressed explicitly as:

(6) $$\begin{array}{c} \varphi_{R}\left(x,y\right)=-\frac{2\pi }{\lambda_{R}}\left(\sqrt{{\left(x-x_{R}\right)}^{2}+{\left(y-y_{R}\right)}^{2}+f^{2}}-f\right)+C_{R},\\ \varphi_{G}\left(x,y\right)=-\frac{2\pi }{\lambda_{G}}\left(\sqrt{{\left(x-x_{G}\right)}^{2}+{\left(y-y_{G}\right)}^{2}+f^{2}}-f\right)+C_{G},\\ \varphi_{B}\left(x,y\right)=-\frac{2\pi }{\lambda_{B}}\left(\sqrt{{\left(x-x_{B}\right)}^{2}+{\left(y-y_{B}\right)}^{2}+f^{2}}-f\right)+C_{B},\\ \varphi_{NIR}\left(x,y\right)=-\frac{2\pi }{\lambda_{NIR}}\left(\sqrt{{\left(x-x_{NIR}\right)}^{2}+{\left(y-y_{NIR}\right)}^{2}+f^{2}}-f\right)+C_{NIR}. \end{array}$$

**Figure 7:**
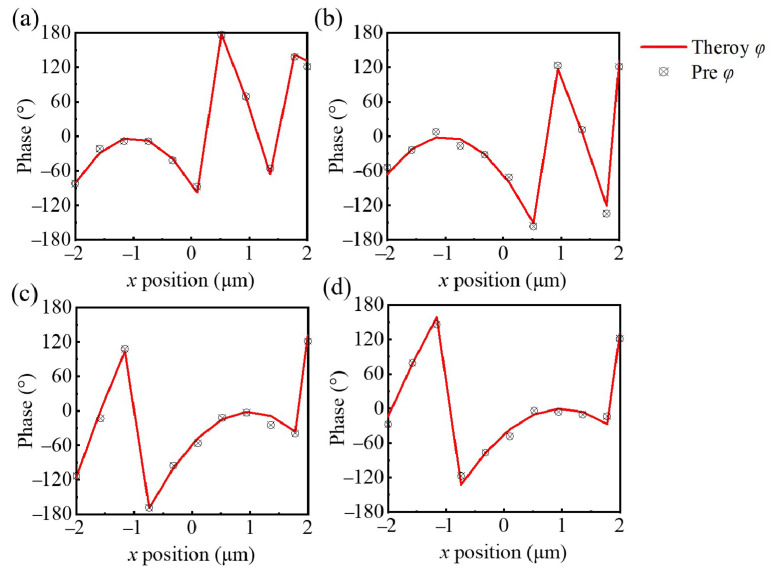
Comparison of the theoretical phase distribution and the phase distribution of the meta-atoms obtained through inverse design at center wavelengths of (**a**) 450 nm, (**b**) 540 nm, (**c**) 630 nm, and (**d**) 800 nm.

In all the equations as mentioned above, $\lambda_{R,G,B,NIR}$ represents the central wavelengths for the different spectra, specifically for red, green, blue, and near-infrared. The variables $x_{R,G,B,NIR}$ and $y_{R,G,B,NIR}$ denote the focal center positions corresponding to each color, while *f* represents the focal length. Additionally, to achieve phase synchronization modulation across all spectral channels and to compensate for errors, a fitting constant $C_{R,G,B,NIR}$ is employed. This value is determined through the iterative optimization of fitting parameters based on several different combinations aimed at minimizing the discrepancy between the actual phase of the meta-atoms and the theoretical phase. During each iteration, the optimization of the meta-atoms was performed using a combination of deep learning and inverse design methods. The final results are presented in Figure 7, which shows the comparison between the theoretical phase distribution and the actual phase distribution at four wavelengths, along with the corresponding transmittance curves. From the figure, it is evident that when the fitting constant $C_{R,G,B,NIR}$ takes values of 0, 1.50, 1.54, and 0.56, the phase error of the meta-atoms is minimized, remaining below 15° in most cases, while the transmittance exceeds 80% for all wavelengths.

**Figure 8:**
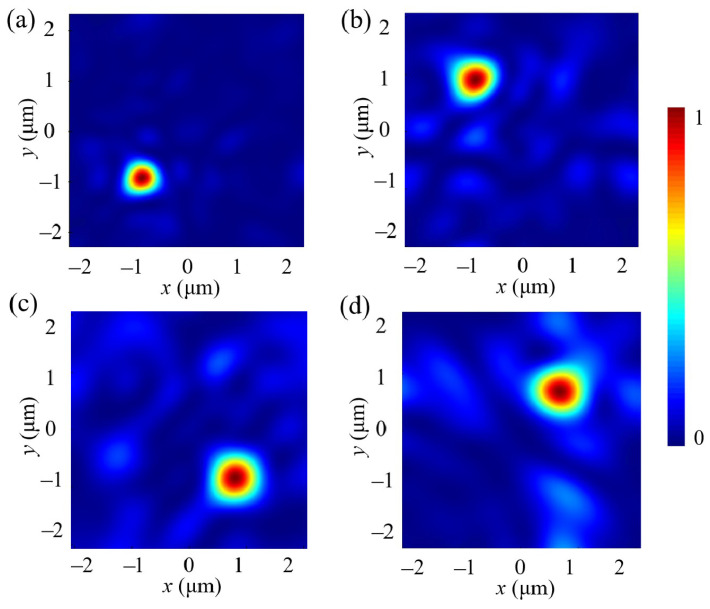
The normalized electric field distributions at center wavelengths of (**a**) 450 nm, (**b**) 540 nm, (**c**) 630 nm, and (**d**) 800 nm.

**Figure 9:**
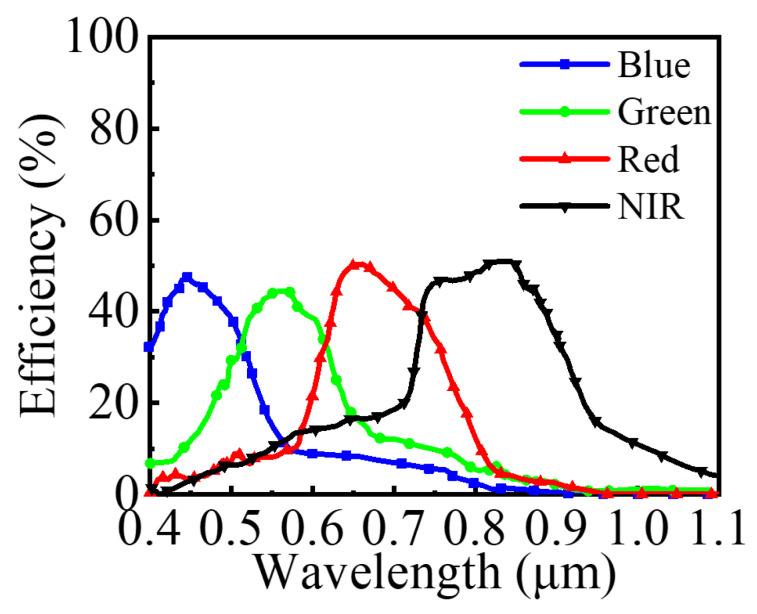
The optical efficiency of the metalens over a broadband spectral range.

Finally, we modeled and simulated the metalens based on the relationship between position and phase. Figure 8 presents the electric field distribution in the focusing cross-section of the metalens at the four wavelengths. The four wavelengths are focused near the target positions, demonstrating the effective routing and focusing capabilities of the designed metalens. To evaluate the optical efficiency for each wavelength, we calculated the integrated power density for each 2 × 2 μm target area, divided by the total optical power incident on the entire metalens. As shown in Figure 9, the optical efficiency curves for the blue (400–500 nm), green (500–600 nm), red (600–700 nm), and near-infrared (700–900 nm) spectral ranges peak within the specified target areas. The average optical efficiencies achieved are 42.15%, 40.51%, 41.62%, and 40.50%, respectively. As summarized and compared in Table 2, the design of the metalens color router presented in this paper is more straightforward and significantly reduces time consumption compared to previous designs. Additionally, it can simultaneously route channels across four wavelength ranges and achieve over 40% optical efficiency within a broad wavelength range. This exceeds the maximum efficiency of 25% that can be achieved by traditional filters using four spectral channels. These results demonstrate the advancement and effectiveness of our proposed scheme, which combines deep learning and particle swarm optimization. Although the pixel size used in this study deviates from the current mainstream fabrication requirements, the same design for the metalens can be achieved through the combination of adjacent pixels. For the metalens we designed, the inclined profile of the vertical sidewalls has a limited impact on the results, with the primary concern being to avoid deviations in structural dimensions. Therefore, advanced nanofabrication techniques, such as deep reactive ion etching (DRIE) and metal-assisted chemical etching (MACE), can be utilized to manufacture such high aspect ratio nanostructures, which will help mitigate the impact of fabrication errors on the results.

## 4. Conclusions

In summary, we propose an inverse design method for a metalens color router based on deep learning networks and particle swarm optimization algorithms. This novel approach provides a rapid means of satisfying wide-wavelength phase distributions, catering to the needs of various metalens applications, including color routers. By training discrete meta-atoms, we can quickly predict their phase and transmittance across the 400–1100 nm wavelength range, achieving high accuracy in phase prediction and transmittance prediction. The transmittance prediction error of the model is mainly within the range of 0 to 1%, with an overall average prediction error of 0.5%. And the speed of predicting a single meta-atom is only 1 ms. The integration of the particle swarm optimization algorithm allows for the rapid identification of high-transmittance meta-atom structures that meet the target phase or phase combinations. The single-wavelength focusing metalens was successfully validated using a combination of deep learning and particle swarm optimization algorithms. Following this, we arranged different meta-atoms according to the pre-designed phase distribution to meet the requirements of the color router. Simulation results demonstrate that the designed meta-lens color router can selectively focus R, G, B, and NIR light into four distinct regions, with efficiencies exceeding 40% for each band, significantly surpassing the efficiency of traditional color filters. The proposed design offers a promising solution for achieving selective light classification across a broad spectral range.

## Figures and Tables

**Table 1:** Comparison with Other Similar Inverse Design Schemes for Metalens.

Reference	Design Scheme (Main Network Architechctuer/ Inverse Algorithm)	Types of Meta-Atom	Wavelength	Predicted Content	Prediction Deviation
[43]	CNN/None	Discrete Pixelated Metasurface	10 GHz	Phase	90%
[44]	MLP/PSO	Cross, Circular Hole, Square Hole Structure	420–640 nm	Phase	Unspecified
[45]	CNN, MLP/GA	Dielectric Free-form Metasurface	400–700 nm	Phase/Transmission	>99%
[46]	MLP/GA	*C*_6-Symmetrical Coding Structure	30 mm	Phase/Reflectance	>88%
This Paper	MLP, CNN, Transform/PSO	Discrete Pixelated Metalens	400–1100 nm	Phase/Reflectance	>99%

**Table 2:** Comparison with previously pioneered routers.

Reference	Material	Types of Meta-Atom	Working Wavelength	Efficiency	Manufacturing Process Difficulty
[47]	Semiconductor	RGB Clear color filter	Not Specifically Mentioned	∼25% (R)\50% (G)\25% (B)	Simple
[5]	Al	Metallic Circular Hole	300–900 nm	38% (R)\38% (G)\28% (B)	Moderate
[7]	Si_3_N_4_	3D metamaterial	400–700 nm	33.9% (R)\56.2% (G)\58.412% (B)	Extremely Difficult
[8]	Si_3_N_4_	Combination of Multiple Unit Structures	400–700 nm	48% (R)\48% (G)\12% (B)	Moderate
[25]	Si_3_N_4_	Combination of Multiple Unit Structures	400–1100 nm	37.1% (R)\28.6% (G)\26.5% (B)\ 39.1% (NIR)	Moderate
[48]	Si_3_N_4_	Freeform Metasurface	400–700 nm	60% (R)\57% (G)\65% (B)	Difficult
This Paper	Si_3_N_4_	Discrete Structure	400–700 nm	42.15% (R)\40.51% (G)\41.62% (B)\40.50% (NIR)	Difficult

## Data Availability

The data that support the findings of this study are available upon reasonable request from the authors.
